# Case report: A novel compound heterozygous variant in the *COL4A3* gene was identified in a patient with autosomal recessive Alport syndrome

**DOI:** 10.3389/fgene.2024.1426806

**Published:** 2024-07-12

**Authors:** Sha Chen, Yufeng Zhang, Jinjin He, Dingwei Yang

**Affiliations:** ^1^ Department of Nephrology, Tianjin Hospital, Tianjin University, Tianjin, China; ^2^ Clinical College of Orthopedics, Tianjin Medical University, Tianjin, China

**Keywords:** Alport syndrome, *COL4A3*, compound heterozygous variant, next-generation sequencing, case report

## Abstract

Alport syndrome (AS), a hereditary kidney disease with a high risk for renal failure, is attributed to pathogenic variants in genes *COL4A3*, *COL4A4*, and *COL4A5* that encode type IV collagen. Next-generation sequencing (NGS) is increasingly applied to the diagnosis of AS, but complex genotype–phenotype correlation, that is, identifying the significance of variants, is still a huge clinical challenge. In this study, we reported the case of a 27-year-old Chinese woman with a family history of hematuria and proteinuria. Notably, the proband is the only one in her family with renal insufficiency. NGS was performed in this family, and it was revealed that the proband was a compound heterozygote for two variants in the *COL4A3* gene: c.2990G>A inherited from her father and c.4981C>T inherited from her mother. We modeled the spatial structure of the corresponding protein and assumed that structural abnormalities led to the breakdown of type IV collagen networks, a major component of the glomerular basement membrane. Thus, the proband was diagnosed with autosomal recessive AS, characterized by severe defects of the glomerular basement membrane. Hence, the proband showed a loss of renal function. This case presentation emphasizes the importance of NGS for AS diagnosis and introduces a novel genotype of AS.

## 1 Introduction

Alport syndrome (AS) is a rare genetic disorder but is also the most common hereditary kidney disease. It is characterized by hematuria, proteinuria, and progressive renal insufficiency, usually accompanied by sensorineural hearing loss and ocular abnormalities (Savige et al., 2022). These symptoms are attributed to pathogenic variants in genes *COL4A3*, *COL4A4*, and *COL4A5* that encode type IV collagen α3-, α4-, and α5-chains, the principal components of basement membranes in the glomerulus, cochlea, and lens (Savige et al., 2022). According to the modes of inheritance, AS is classified into four types: X-linked AS caused by pathogenic variants in the *COL4A5* gene; autosomal recessive AS (ARAS) caused by homozygous or compound heterozygous variants in the *COL4A3* or *COL4A4* gene; autosomal dominant AS (ADAS) caused by heterozygous variants in the *COL4A3* or *COL4A4* gene; and digenic AS caused by coexisting variants in two of the genes *COL4A3*, *COL4A4*, and *COL4A5* (Kashtan, 2021). Next-generation sequencing (NGS), which can detect variants, is an efficient tool for the diagnosis of AS (Moriniere et al., 2014). However, with the detection of an increasing number of novel variants, the identification of pathogenic genotypes has become another major clinical challenge (Kashtan, 2021).

In this study, we reported the case of a young female patient with a family history of hematuria and proteinuria. Unfortunately and confusingly, this patient is the only one with renal insufficiency, which eventually progressed into end-stage renal disease (ESRD) in her family. This patient was ultimately diagnosed with ARAS by NGS, and a novel severe genotype, compound heterozygote for *COL4A3* c.2990G>A and *COL4A3* c.4981C>T, was identified.

## 2 Case description

### 2.1 Clinical history and laboratory examination results

A 27-year-old Chinese woman with no significant past medical history was referred to our nephrology department due to complaints of hematuria, proteinuria, and renal insufficiency. At age 20, she had edema in the lower extremities, and the laboratory tests indicated sub-nephrotic range proteinuria and mild persistent hematuria with a normal serum creatinine (Scr) level. At age 25, her level of Scr was found to be elevated to more than 110 µmol/L and then increased gradually. No hearing loss or ocular lesions were observed in this patient. Chronic glomerulonephritis was diagnosed initially, and thunder god vine, glucocorticoid, and decoction were prescribed to her during the course of the disease. However, there was no significant improvement, and drug-related toxic effects were observed. Until she was hospitalized in our department, her Scr level had increased to 790.8 µmol/L, and an ultrasound revealed that both kidneys had become atrophic, indicating that she had progressed to ESRD. Her physical examination findings were unremarkable. Urinalysis on admission revealed proteinuria (3+) and hematuria (141 red blood cells/μL), and 24-h urinary protein excretion was 2.068 g/24 h. Kidney function tests indicated that the Scr level was 790.8 μmol/L and the estimated glomerular filtration rate was 5.46 mL/min/1.73 m^2^. Laboratory examinations of autoantibodies (such as antinuclear antibodies and antineutrophil cytoplasmic antibodies), immunoglobulin, light chains, and so on showed no abnormalities.

### 2.2 Familial history

**FIGURE 1:**
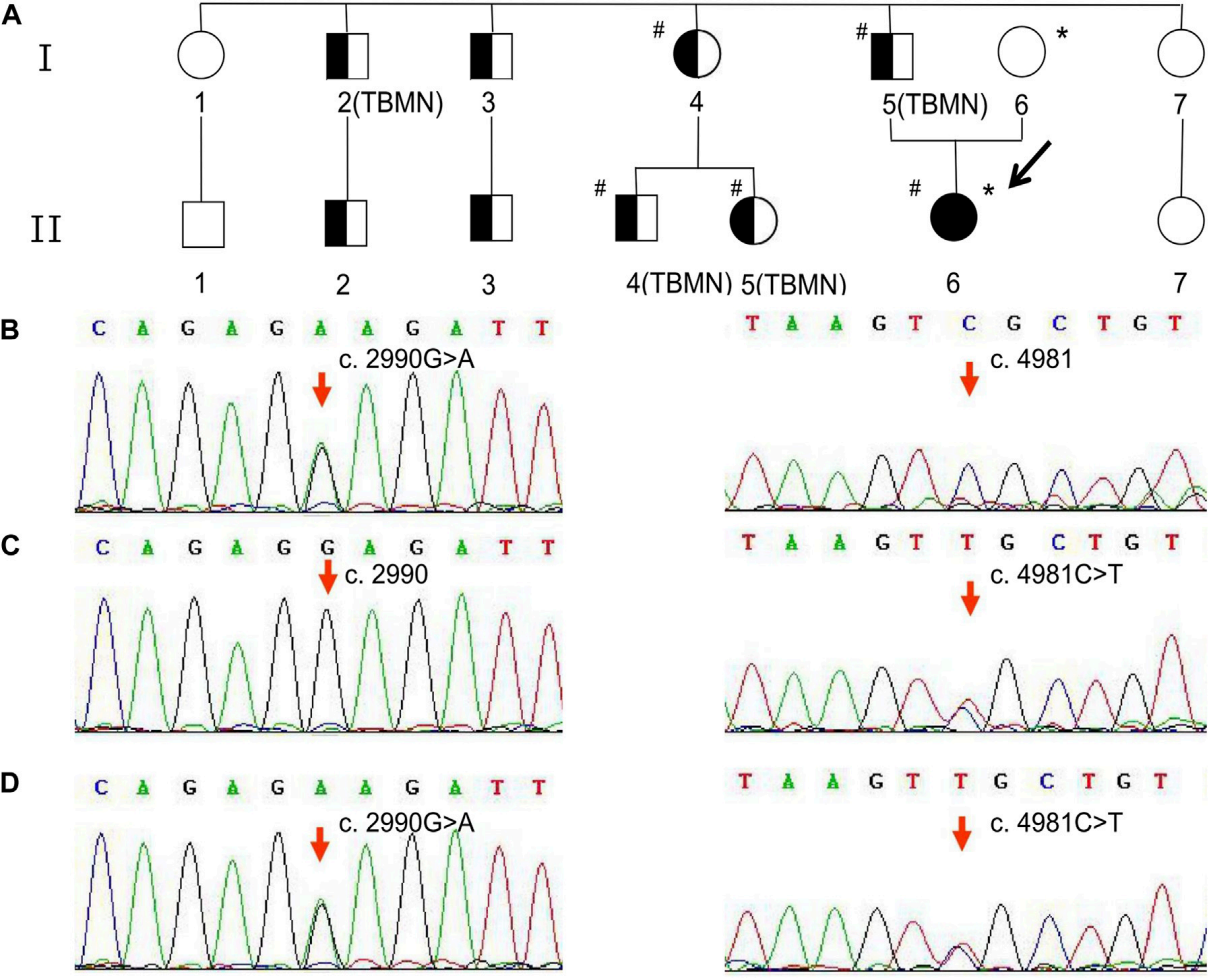
Family pedigree and variant analysis of the *COL4A3* gene. **(A)** The full-shaded icon denotes end-stage renal disease; half-shaded icons denote individuals with microscopic hematuria and proteinuria but with normal renal function. The pound sign (#) indicates the individual with the *COL4A3* c.2990G>A (p.Gly997Glu) variant. The asterisk (*) indicates the individual with the *COL4A3* c.4981C>T (p.Arg1661Cys) variant. TBMN indicates the individuals whose renal pathological findings reveal uniform thinning of the glomerular basement membrane. **(B)** The proband’s father and patrilineal relatives who underwent genetic testing were detected to have a heterozygous variant c.2990G>A. **(C)** The proband’s mother was an asymptomatic carrier of heterozygous variant c.4981C>T carrier. **(D)** The proband harbored a compound heterozygous variant, c.2990G>A and c.4981C>T.

Notably, this patient had a significant family history of abnormal urinalysis and is referred here as the proband (II6). Nine affected members of the family, including the proband and her father, presented with symptoms of hematuria and proteinuria (Figure 1A). Among them, four individuals (including the proband’s father) underwent a renal biopsy, and all of their pathological findings revealed uniform thinning of the glomerular basement membrane (GBM) (Figure 1A). The urinalysis and renal function of the proband’s mother were normal. Confusingly, the proband was the only one who showed renal insufficiency among the affected members of her family. To clarify this, genetic testing was recommended for the proband and her family members.

### 2.3 Genetic testing

The proband, her parents, and some affected members consented to NGS. Following the collection of 2-mL peripheral blood samples from the proband and family members, genomic DNA was extracted using a QIAsymphony DSP DNA Mini Kit (QIAGEN, Germany), according to the manufacturer’s protocol. The concentration and purity of DNA were measured using a NanoDrop 2000 ultra-microspectrophotometer (Thermo Scientific, United States). The total exon library was constructed by DNA fragmentation, end-repair, A-tailing, adapter ligation, hybridization and capture of DNA library samples, and PCR amplification. Whole-exome sequencing, one of the NGS techniques, was carried out at KingMed Diagnostics (Tianjin, China) with a NovaSeq 6000 sequencer (Illumina, United States). The sequencing depth of the target area was more than 20× with 98% coverage. The obtained DNA sequences were aligned to the reference human genome (UCSC hg19) using Burrows–Wheeler Aligner software. GATK software was used for bioinformatics analysis. Sanger sequencing was used to confirm the identified variants.

### 2.4 The results of NGS

As shown in Figure 1B, the heterozygous variant, c.2990G>A (NM_000091.5) in exon 36 of the *COL4A3* gene, was detected in the proband’s father and patrilineal relatives who underwent genetic test (I4, I5, II4, and II5). The missense variant (*COL4A3* c.2990G>A) caused a substitution of glycine by glutamic acid at amino acid position 997 (p.Gly997Glu). Another heterozygous missense variant, c.4981C>T (NM_000091.5; p.Arg1661Cys), was identified in exon 52 of the *COL4A3* gene in the proband’s mother (Figure 1C). The proband inherited both variants from her parents (Figure 1D). According to the classification criteria of the American College of Medical Genetics and Genomics (ACMG) for the clinical significance of genetic variants, *COL4A3* c.2990G>A and *COL4A3* c.4981C>T are both classified as “likely pathogenic”. The variation data reported in this paper have been deposited in the Genome Variation Map (GVM) in National Genomics Data Center, Beijing Institute of Genomics, Chinese Academy of Sciences and China National Center for Bioinformation, under accession number GVM000739.

### 2.5 Diagnosis, treatment, and outcome

Based on the family history, symptoms, and laboratory findings, ARAS was diagnosed in the proband. Due to a severe loss of renal function, she underwent maintenance hemodialysis. At age 28, a kidney transplantation was performed successfully. She then stopped renal replacement therapy and was lost to follow-up.

## 3 Discussion

The proband presented with hematuria, proteinuria, and renal dysfunction. Glomerulonephritis was initially considered, but she failed to respond to immunosuppressive therapy and presented with a rapid progression of nephropathy. Unfortunately, she was unable to undergo a renal biopsy after admission due to renal atrophy, which increased the difficulty of the diagnosis. Due to her particular family history, NGS was conducted, which showed the presence of two variants.

The c.2990G>A (p.Gly997Glu) variant of the *COL4A3* gene, one of the proband’s variants, was inherited from her father. All patrilineal relatives of the proband with hematuria and proteinuria, regardless of age or sex, had normal renal function. Although not all affected individuals underwent renal biopsy and genetic tests, the available results of the examinations indicated uniform thinning of the GBM and the heterozygous variant of c.2990G>A in the *COL4A3* gene. The uniformly thin GBM induced by defective synthesis of type IV collagen, previously referred to as “thin glomerular basement membrane nephropathy,” is caused by heterozygous variants in the *COL4A3* or *COL4A4* genes and is usually associated with a better prognosis (Hirabayashi et al., 2022). In recent years, considering that the thin basement membrane may be an early pathological manifestation of ADAS, the term “thin glomerular basement membrane nephropathy” has been gradually replaced by “type IV collagen-related disease” (Comic et al., 2022) or “*COL4A3* or *COL4A4* heterozygotes” (Savige et al., 2022). Three individuals with a heterozygous variant of *COL4A3* c.2990G>A in one family were detected in a previous study (Lin et al., 2014). Of these, two individuals had clinical manifestations similar to those of the affected members in the proband’s family, but the third member presented with progressive renal impairment and developed ESRD at age 55 (Lin et al., 2014). The dramatic phenotypic differences may be related to the presence of coexisting diseases (e.g., diabetes and hypertension), lifestyle, and so on. However, the pathogenicity of this variant, *COL4A3* c.2990G>A, in type IV collagen-related diseases is clear.

Unlike the *COL4A3* c.2990G>A variant, the proband’s other variant the, *COL4A3* c.4981C>T (p.Arg1661Cys), did not cause any symptoms of kidney disease in her mother, which is consistent with the studies conducted by Sienes et al. (2021) and Braunisch et al. (2018). Two cases, a 38-year-old male patient and a 34-year-old female patient, diagnosed with ARAS caused by compound heterozygous variants in the *COL4A3* gene, were reported by Sienes et al. and Braunisch et al., respectively. Both patients inherited the same variant, *COL4A3* c.4981C>T (p.Arg1661Cys), from their mothers, and both mothers were asymptomatic. In addition, their fathers showed normal kidney function. However, unlike our proband, these two patients had hearing impairment, which may be related to the difference in another gene variant. To some extent, these studies revealed the pathogenicity of the *COL4A3* c.4981C>T variant in ARAS. Although given that all patients’ mothers did not have hematuria, there is another possibility that this variant only plays a modifying role and the other variant is responsible for the clinical presentation of AS, but the phenotypic differences between patients and their fathers and even other family members further support the important role of the *COL4A3* c.4981C>T variant in the genetic diagnosis of ARAS. However, this still requires more clinical data or animal studies to provide sufficient evidence.

**FIGURE 2:**
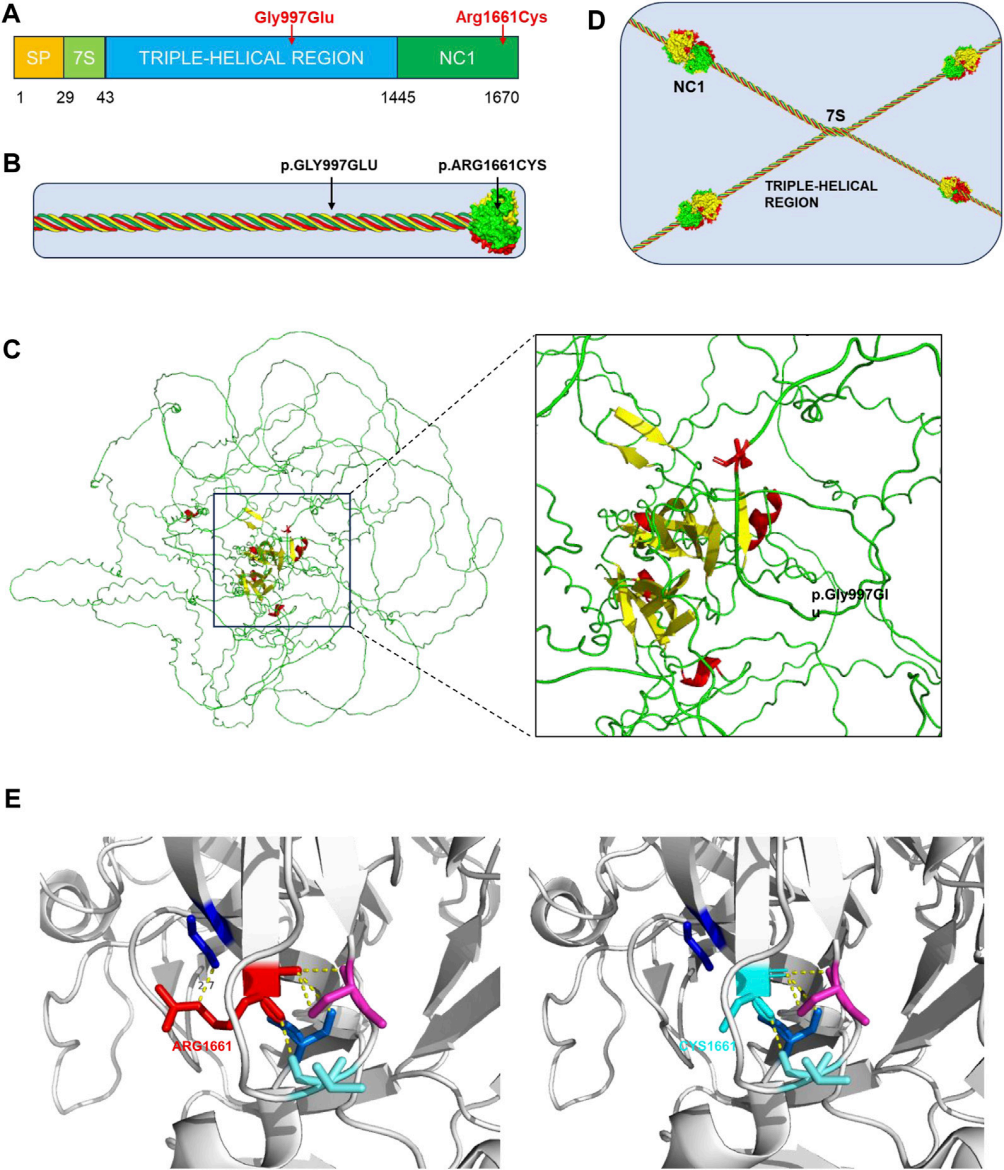
Type IV collagen, type IV collagen α3-chain, collagen IV reticulum, and presentation of p. Gly997Ser and p. Arg1661Cys mutants. **(A)** The collagen α3 (IV) chain contains a short triple-helical structural domain (7S) in the N-terminal region, a central long triple-helical structural domain and a C-terminal non-collagenous structural domain (NC1), and the gene variant p.Gly997Glu is located in the central triple-helical collagenous region, whereas the variant p.Arg1661Cys is located in the NC1 structural domain. **(B)** Location of the gene variant site in the α3α4α5 triple helix type IV collagen monomer. **(C)** 3D representation of the *COL4A3* mutant of p.Gly997Glu (AF-Q01955-F1, AlphaFold), in which Gly997 was replaced with Glu, whose larger amino acid residues affect the stability of the collagen structural domains. **(D)** Basic structure of type IV collagen. Each type IV collagen monomer is bridged to the NC1 hexamer by NC1 trimer-trimer binding. The four 7S structural domains are linked together by end-to-end oligomerization to form a tetramer, which binds to each other to form reticular structure. **(E)** 3D representation of the *COL4A3* mutant of p.Arg1661Cys (PDB 6WKU, PdbViewer), whose Arg1661 was replaced with an uncharged Cys, with alterations in hydrogen bonding patterns, hydrophobic forces, and electrostatic interactions affecting the structural stability of type IV collagen.

The genetic variants alter the normal structure of type IV collagen and, in turn, that of the GBM, which is the main pathogenesis of AS. Each α-chain in type IV collagen molecules consists of three domains: the N-terminal 7S domain, the collagenous domain, and the C-terminal NC1 domain (Figure 2A) (Suh and Miner, 2013). The collagenous domain is characterized by a triple helix that comprises many Gly-X-Y repeat sequences (Miner, 1999). Glycine with only one hydrogen residue is smaller than the other amino acids and reduces steric hindrance to fit into the center of the triple-helical structure. The c.2990G>A variant in our proband induces a glycine substitution and disrupts the stability of the triple helix (Figures 2B, C). Another variant, c.4981C>T (Arg1661Cys), is located in the NC1 domain (Figure 2B) and is responsible for the connection between type IV collagen molecules (Figure 2D). The substitution of uncharged cysteine for positively charged arginine results in changes in hydrogen bonds, hydrophobic forces, and electrostatic interactions on the one hand and the possible formation of new disulfide bonds on the other hand (Figure 2E). Consequently, the normal architecture of the NC1 domain is disrupted. All of these changes lead to defects in the type IV collagen network. Corresponding to one copy of the *COL4A3* gene with the variant, the proband’s parents have another normal copy to compensate. In the proband, however, both copies are affected. The absence of a normal α3-chain and the increased proportion of abnormal collagens are the reasons why the phenotype of the proband is more severe than that of her parents. Although there were no proven mutational “hotspots” in the *COL4A3* or *COL4A4* genes, the coexisting variant of a glycine deletion in the collagenous domain and a cysteine substitution in the NC1 domain has been increasingly observed in patients with ARAS (Storey et al., 2013).

Considering clinical manifestations, laboratory findings, and especially genetic testing results, the proband was diagnosed with ARAS. Her poor prognosis is consistent with the observations in previous studies. The phenotypes and outcomes are largely dependent on the genotypes of AS patients. It was reported that the median age of progression to ESRD in ARAS patients was 21 years (Oka et al., 2014), but that of renal insufficiency in ADAS patients was 70 years (Nozu et al., 2019). Although there is currently no effective cure for AS, many previous studies have verified that the timely initiation of angiotensin-converting enzyme inhibitor medication confers a significant benefit in delaying progression to ESRD (Zhang et al., 2016; Kashtan, 2021; Mastrangelo et al., 2021; Boeckhaus et al., 2022; Gregorio et al., 2023), especially before impairment of renal function (Rheault, 2020; Kashtan, 2021). Unfortunately, the proband had already missed the opportunity to take angiotensin-converting enzyme inhibitor medication and progressed to ESRD at the age of only 27 years. Thus, early diagnosis and prompt treatment of AS are crucial. Recently, NGS has been increasingly used in diagnosing AS due to its sensitivity and non-invasiveness. However, the high cost and indefinite significance of variants prevent the widespread use of NGS. The identification of the compound heterozygous variants of *COL4A3* c.2990G>A and *COL4A3* c.4981C>T in this case report expands the genotypic spectrum of ARAS. In addition, this case report reveals the need for NGS testing in individuals from families with hereditary kidney disease. It not only facilitates the early detection of AS and pathogenetic variants but also contributes to eugenics, that is, avoiding the birth of neonates with a high risk of AS.

However, this study has some limitations. First, there was only one case in this study, resulting in a lack of information regarding genotype–phenotype correlations. Second, further *in vitro* and *in vivo* studies are needed to confirm the pathogenicity of the variants and their underlying mechanisms.

In conclusion, the presented case report emphasizes the importance of genetic testing in individuals with a suspicion of AS. It not only assists in the diagnosis but also reveals the location and type of variant, helping to choose the appropriate treatment and predict the prognosis. More importantly, a novel combination of pathogenic variants in ARAS, the compound heterozygous variants of *COL4A3* c.2990G>A and *COL4A3* c.4981C>T, was identified in this case. This helps understand the genotype–phenotype correlation and thereby benefits the genetic diagnosis and counseling of AS.

## Data Availability

The data presented in the study are deposited in the Genome Variation Map (GVM) repository in National Genomics Data Center, Beijing Institute of Genomics, Chinese Academy of Sciences and China National Center for Bioinformation, accession number GVM000739. The Link as follows: https://ngdc.cncb.ac.cn/gvm/getProjectDetail?project=GVM000739.
